# 76 Implementation of a Mupirocin Prophylaxis Policy for Reduction of Transmission of MRSA

**DOI:** 10.1093/jbcr/iraf019.076

**Published:** 2025-04-01

**Authors:** Emily Werthman, Julie Caffrey, Geeta Sood, Matthew Heron

**Affiliations:** Johns Hopkins Burn Center; Johns Hopkins University School of Medicine; Johns Hopkins Medicine; Johns Hopkins University School of Medicine

## Abstract

**Introduction:**

Infections carry high morbidity and mortality for patients with burns. Rapid testing for methicillin-resistant Staphylococcus aureus (MRSA) nasal colonization is common practice in many burn intensive care units (BICUs), but there is minimal recent evidence supporting MRSA prophylaxis. Topical mupirocin is inexpensive, low-risk, and may reduce complications secondary to MRSA infection. This study aimed to determine the impact of prophylactic intranasal mupirocin on the incidence of bacterial infections in one BICU.

**Methods:**

In March 2021, our BICU implemented the prophylactic administration of mupirocin to the bilateral nares of all admitted patients. To assess the efficacy of this intervention, we conducted a retrospective review of all patients admitted to our BICU between January 1, 2017, and December 31, 2022. We divided patients into two cohorts: pre-mupirocin (i.e., those receiving care before March 1, 2021) and post-mupirocin (i.e., those receiving care on or after March 1, 2021). We then reviewed inpatient microbiology results, including blood and respiratory cultures, to identify differences in the incidence of bacterial infections.

**Results:**

We identified 896 total patients. There were 699 patients in the pre-mupirocin cohort and 197 patients in the post-mupirocin cohort. There were no significant differences between groups at baseline with respect to age, arrival carboxyhemoglobin, arrival Glasgow Coma Scale score, and the average total body surface area (TBSA) with second- or third-degree burns. There were significantly more cases of culture-proven MRSA (n=12) and methicillin-sensitive staphylococcus aureus (MSSA, n = 9) in the pre-mupirocin cohort than in the post-mupirocin cohort (n = 0 and n =2, respectively). There were also more cases of non-Staphylococcal infections in the pre-mupirocin cohort than in the post-mupirocin cohort.

**Conclusions:**

The introduction of prophylactic intranasal mupirocin for patients with burns was associated with reductions in the incidence of MRSA, MSSA, and other bacterial infections in one BICU.

**Applicability of Research to Practice:**

Protocols including the routine application of intranasal mupirocin may alleviate patient morbidity and mortality in BICU settings.

**Funding for the Study:**

N/A

